# Occurrence of third-generation cephalosporin-resistant *Escherichia coli* in European hedgehogs *(Erinaceus europaeus)* from a wildlife rescue centre in Lombardy, Northern Italy

**DOI:** 10.1007/s11259-026-11356-4

**Published:** 2026-06-20

**Authors:** S. Raineri, A. Gazzola, G. Dilio, S. Ventura, A. M. Maisano, F. Guarneri, N. Formenti, G. L. Alborali, C. F. Magistrali, J. Filipe, Maria Cristina Rapi, G. Grilli

**Affiliations:** 1 Istituto Zooprofilattico Sperimentale della Lombardia e dell’Emilia Romagna ’Bruno Ubertini’ (I.Z.S.L.E.R.), Via Bianchi 7/9, Brescia, 25124 Italy; 2 Department of Veterinary Medicine and Animal Sciences, University of Milan, Via dell’Università, 6, Lodi, 26900 Italy; 3 Laboratorio di Malattie Infettive degli Animali (MiLab), University of Milan, Via dell’Università, 6, Lodi, 26900 Italy; 4 Wildlife Health Lab (WHL), University of Milan, Via dell’Università, 6, Lodi, 26900 Italy

**Keywords:** Antimicrobial resistance, 3GCR-*E. coli*, One health, European hedgehogs

## Abstract

Hedgehogs represent a promising indicator for evaluating environmental health. We investigated the occurrence of third-generation cephalosporin-resistant (3GCR) *Escherichia coli* in intestinal contents from 49 European hedgehogs (*Erinaceus europaeus*) deceased at a Wildlife Rescue Centre in Lombardy, Northern Italy. Bacteriological analysis was performed to isolate 3GCR-*E. coli*; antimicrobial-resistance (AMR) profiles of the isolates were evaluated by broth dilution (MIC). The presence of *bla*_CTX−M,_ *bla*_TEM_, *bla*_SHV_, *bla*_CMY−2_genes was assessed by PCR assay. 3GCR-*E. coli* were detected in 51% of the examined hedgehogs (*n* = 25/49). The majority of the isolates were multidrug resistant (MDR); resistance was recorded against the β-lactams cefotaxime, cefazolin, and ampicillin in 100% (*n* = 25/25) of isolates while amoxicillin/clavulanic acid in 52% (*n* = 13/25). Resistance against enrofloxacin and flumequine was found in 92% (*n* = 23/25) of isolates and tetracycline in 64% (*n* = 16/25). All the isolates were susceptible to colistin and florfenicol, and most of them were susceptible to kanamycin (96%, *n* = 24/25). The most frequently detected resistance gene was *bla*_TEM_ (52%, *n* = 13/25), followed by *bla*_SHV_ (36%, *n* = 9/25) and *bla*_CTX−M_ (28%, *n* = 7/25). Seven isolates carried two ESBL genes, five being *bla*_TEM_ / *bla*_SHV_, and two *bla*_TEM_ / *bla*_CTX−M_. No isolates harbored *bla*_CMY−2_ gene. Our study provides the first data on the occurrance of 3GCR-*E. coli* in European hedgehogs admitted to a wildlife rescue centre in Northern Italy. However, since the sampled animals were not collected through a field-based survey, the findings should not be considered representative of the wider free-ranging population. Nonetheless, resistance profiles and genomic characterization of these isolates provide useful information on AMR circulation in human-impacted environments.

**Supplementary Information:**

The online version contains supplementary material available at https://doi.org/10.1007/s11259-026-11356-4.

## Introduction

Antimicrobial resistance (AMR) is an increasing global concern, characterized by microorganisms acquiring physical and biochemical mechanisms that render antimicrobial agents ineffective (Abushaheen et al. 2020; Caneschi et al. 2023). This loss of efficacy of antiviral, antiparasitic, antifungal, and antibiotic treatments complicates infection management and, in some cases, renders infection untreatable. The socio-economic burden of AMR, including increased healthcare costs and rising mortality rates, has positioned it among the top ten threats to human, animal, and environmental health in the 21st century (Huemer et al. 2020; Murray et al. 2022). Although AMR is a naturally occurring phenomenon, the inappropriate use of antimicrobials in human and veterinary medicine has accelerated this process. Resistance-conferring genes (ARGs) originally evolved as a defense against natural antimicrobial compounds produced by bacteria, fungi, or plants. However, human-driven antimicrobial misuse has intensified selective pressure within microbial communities, thus accelerating AMR development (Page and Gautier 2012; Llor and Bjerrum 2014; Caneschi et al. 2023). The ability of ARGs to spread among microorganisms via horizontal transfer (HGT) further exacerbates the crisis by facilitating the widespread distribution of resistance determinants across bacterial populations (Allen et al. 2010; Holmes et al. 2016; Von Wintersdorff et al. 2016).

Given the current standstill in the development of novel antimicrobial agents and the increasing prevalence of bacteria resistant to last-resort antimicrobials (Wellington et al. 2013), comprehensive AMR surveillance has become more urgent than ever. In this context, the isolation of antimicrobial-resistant bacteria (ARB) from wildlife has garnered increasing scientific interest (Laborda et al. 2022). Although wild animals are not usually directly exposed to antimicrobial agents, the use of these compounds in human and veterinary medicine indirectly influences the emergence and occurrence of ARB in free-living species (Radhouani et al. 2014; Vittecoq et al. 2016; Caneschi et al. 2023). Indeed, it is well established that ARB are widely distributed in natural ecosystems (Laborda et al. 2022; Depta and Niedźwiedzka-Rystwej 2023), primarily due to anthropogenic contamination (Sayah et al. 2005; Swift et al. 2019). Additionally, AMR in wild species may also result from the direct transmission of ARB from pets, livestock, and humans (Karesh et al. 2012; Vittecoq et al. 2016). As a result, animals living near anthropized areas and having frequent human contact are more prone to harbour ARB in their gut compared to those living in habitats characterized by a low human footprint (Skurnik et al. 2006; Kozak et al. 2009; Guenther et al. 2011; Laborda et al. 2022). Consequently, wildlife species are increasingly recognized as valuable bioindicators of environmental AMR contamination (Carroll et al. 2015; Laborda et al. 2022).

Birds and small wild mammals are widely recognized as key bioindicators of environmental AMR, thanks to their ethological, ecological, and feeding-behavior features (Allen et al. 2010; Radhouani et al. 2014; Vittecoq et al. 2016; Furness et al. 2017). Notably, the European hedgehog (*Erinaceus europaeus*) appears to be particularly susceptible to anthropogenic sources of AMR in the environment: the high adaptive capacity, reduced home range and generalist diet of this species make it an effective sentinel for assessing the spread of AMR even at a small spatial scale (Darwich et al. 2019; Garcias et al. 2021). Furthermore, the presence of European hedgehogs in heavily populated areas significantly increases the likelihood of interactions with humans and domestic animals, heightening the opportunities for potential exchange of ARB (Darwich et al. 2019; Rasmussen et al. 2019; Garcias et al. 2021). However, despite growing evidence of AMR circulation in wildlife, data specifically addressing urban-dwelling small mammals remain limited. In particular, the role of European hedgehogs as carriers and potential sentinels of third-generation cephalosporin-resistant (3GCR) *Escherichia coli* in highly urbanized environments has not been fully elucidated.

To address this gap, the present study aims to investigate the presence and frequency of 3GCR-*E. coli* in European hedgehogs (*Erinaceus europaeus*) admitted to a wildlife rescue centre in the Lombardy region (Northern Italy). Considering their restricted home range, close proximity to human settlements, and prolonged exposure to anthropogenic pressures, hedgehogs may represent a locally relevant but still underexplored source of information on AMR circulation. This research therefore aimed to provide further insight into resistance patterns in this study population and their possible relevance in human-impacted environments.

## Materials and methods

### Sample collection

Between October 2023 and January 2024, a total of 49 European hedgehogs that had died naturally or were humanely euthanized were collected from a wildlife recovery center in Northern Italy. Each year, it receives individuals from over half of the provinces in Lombardy, covering approximately 70% of the regional territory. Euthanasia procedures are carried out exclusively under the responsibility of the center’s veterinary director, in compliance with national animal welfare regulations, and only when dictated by severe clinical conditions, major injuries, or when release back into the wild was not feasible. No animals were purposely euthanized for the aims of this study. Only hedgehogs that died prior to receiving any pharmacological or antimicrobial treatment were included in this study. Animals originated from provinces characterized by high human population density and anthropogenic pressure; however, no formal classification of urbanization based on land-use or spatial metrics was applied.

The deceased animals were frozen on-site and transported to the Department of Veterinary Medicine and Animal Sciences, University of Milan, as part of the regional wildlife monitoring program. The cause of admission to the wildlife rescue center, geographical coordinates of the locations where animals were found, as well as the date of admission and death, were recorded and provided by the Wildlife Rescue Centre staff for each of the enrolled hedgehogs.

### Microbiological analysis

All the enrolled animals underwent a complete necropsy, during which intestinal content was collected. Each sample was then enriched at a 1:10 ratio in Buffered Peptone Water (ThermoFisher Scientific, Waltham, MA, United States), and incubated aerobically at 37 °C for 18–24 h. Each enriched sample was then plated onto both MacConkey Agar (ThermoFisher Scientific, Waltham, MA, United States) and CHROMAgar™ ESBL (CHROMAgar™, Paris, France), and incubated aerobically at 37 °C for 18–24 h. MacConkey agar was used as a non-selective growth control for *Enterobacterales*, in order to verify bacterial viability and the presence of lactose-fermenting Gram-negative bacteria in the samples. In contrast, CHROMAgar ESBL was used as a selective medium for the isolation of 3GCR-*E. coli* isolates. For this reason, bacterial isolates were collected exclusively from CHROMAgar ESBL plates. The suspected colonies grown on CHROMAgar™ ESBL plates were identified as *E. coli* using MALDI-TOF mass spectrometry with the MBT Microflex LT/SH MALDI-TOF mass spectrometer (Bruker Daltonik GmbH, Bremen, Germany), following the procedure described by Rosa et al. (2022). One 3GCR-*E. coli* isolate per sample was selected, if present, for antimicrobial susceptibility testing and molecular analysis.

### Antimicrobial susceptibility testing of 3GCR-*E. coli* isolates

Each 3GCR-*E. coli* isolate underwent antimicrobial susceptibility test by broth dilution (EUCAST, 2024; Zimmer 2024) as follows. For each *E. coli* isolate, few colonies were suspended into 3 mL of physiological saline to achieve a turbidity equivalent to the 0.5 McFarland standard (1.5 × 10⁸ CFU/mL), which was verified using a nephelometer. After vortexing, 50 µL of the bacterial suspension were transferred into a tube containing 11 mL of Mueller-Hinton Broth. A custom microdilution plate for Gram-negative enteric pathogens (ThermoFisher Scientific, Waltham, MA, United States), containing a panel of antibiotics commonly used in veterinary and human medicine, was then inoculated by transferring 100 µL of bacterial suspension per well. The plate included β-lactams (amoxicillin/clavulanic acid, ampicillin, cefazolin, cefotaxime), aminoglycosides (gentamicin, kanamycin), fluoroquinolones (enrofloxacin, flumequine), sulphonamides (sulfisoxazole, trimethoprim/sulfamethoxazole), tetracyclines (tetracycline), polymyxins (colistin), and phenicols (florfenicol), providing a broad-spectrum evaluation of resistance patterns in the tested 3GCR-*E*. *coli* isolates. After incubation at 37 °C ± 2 °C for 18 ± 2 h, the plates were analyzed using a semi-automated digital MIC reading system (Thermo Scientific™ Sentititre™ Vizion™ Digital MIC Viewing System).

Bacterial susceptibility to each antimicrobial tested was classified as susceptible (S) or resistant (R). MIC values were interpreted using EUCAST epidemiological cut-off values (ECOFFs) for *Escherichia coli*, retrieved from the EUCAST database (EUCAST 2025). These values were used to distinguish wild-type from non-wild-type isolates. For sulfisoxazole, CLSI M100 (Clinical and Laboratory Standards Insitute 2025) criteria were applied, as no EUCAST ECOFF was available. For the purpose of this study, non-wild-type isolates were categorized as resistant (R), and all results were harmonized into a binary susceptible/resistant classification for ecological and comparative purposes.

MDR bacteria were classified as those resistant to at least one antimicrobial agent in three or more distinct classes of antimicrobials (Magiorakos et al. 2012).

### Molecular characterization of antimicrobial resistance genes (ARGs)

DNA was extracted from the bacterial cultures using the DNeasy Blood and Tissue kit (Qiagen), following the manufacturer’s instructions, and then stored at -20 °C.

The presence of *bla*_CTX-M_, *bla*_TEM_, *bla*_SHV_, and *bla*_CMY-2_ genes was investigated using protocols previously reported in literature (Fang et al. 2008; Rehman et al. 2019). In detail, a multiplex PCR was performed on a Master cycler Nexus X2 (Eppendorf SE, Hamburg, Germany), under the following conditions: an initial denaturation step was run at 95 °C for 15 min, followed by 30 PCR cycles of denaturation at 95 °C for 30 s, annealing at 59 °C for 30 s, elongation at 72 °C for 60 s, with a final extension at 72 °C for 7 min.

All amplicons were analyzed through capillary electrophoresis using a QIAxcel Advanced Instrument (Qiagen, Hilden, Germany) with the QIAxcel ScreenGel Software (Qiagen, Hilden, Germany). The PCR assays were used as a screening tool for the detection of the investigated resistance targets. For *bla*_CTX-M_, *bla*_TEM_, and *bla*_SHV_, the results were interpreted at the gene-family level, and no variant-level characterization was performed. Positive PCR amplicons were not sequenced.

### Statistical analysis

Descriptive statistics were used to summarize the data. The frequency of 3GCR-*E. coli* was calculated as the proportion of positive samples among the total tested, and 95% confidence intervals (95% CI) were computed using the exact binomial method. AMR frequencies were expressed as percentages. MIC distributions were described by reporting the full range of observed values, and MIC₅₀ and MIC₉₀ were determined as the lowest concentrations inhibiting the visible growth of 50% and 90% of isolates, respectively.

Associations between resistance phenotypes were evaluated using Spearman’s rank correlation coefficient (ρ), given the non-parametric distribution of MIC values. Correlation strength was interpreted as weak (ρ < 0.4), moderate (0.4–0.7), or strong (> 0.7). Statistical significance was set at *p* < 0.05. All statistical analyses were performed using Microsoft Excel (Microsoft Corporation, Redmond, WA, USA). Given the exploratory nature of the analysis and the limited sample size, no correction for multiple comparisons was applied.

## Results

**Fig. 1:**
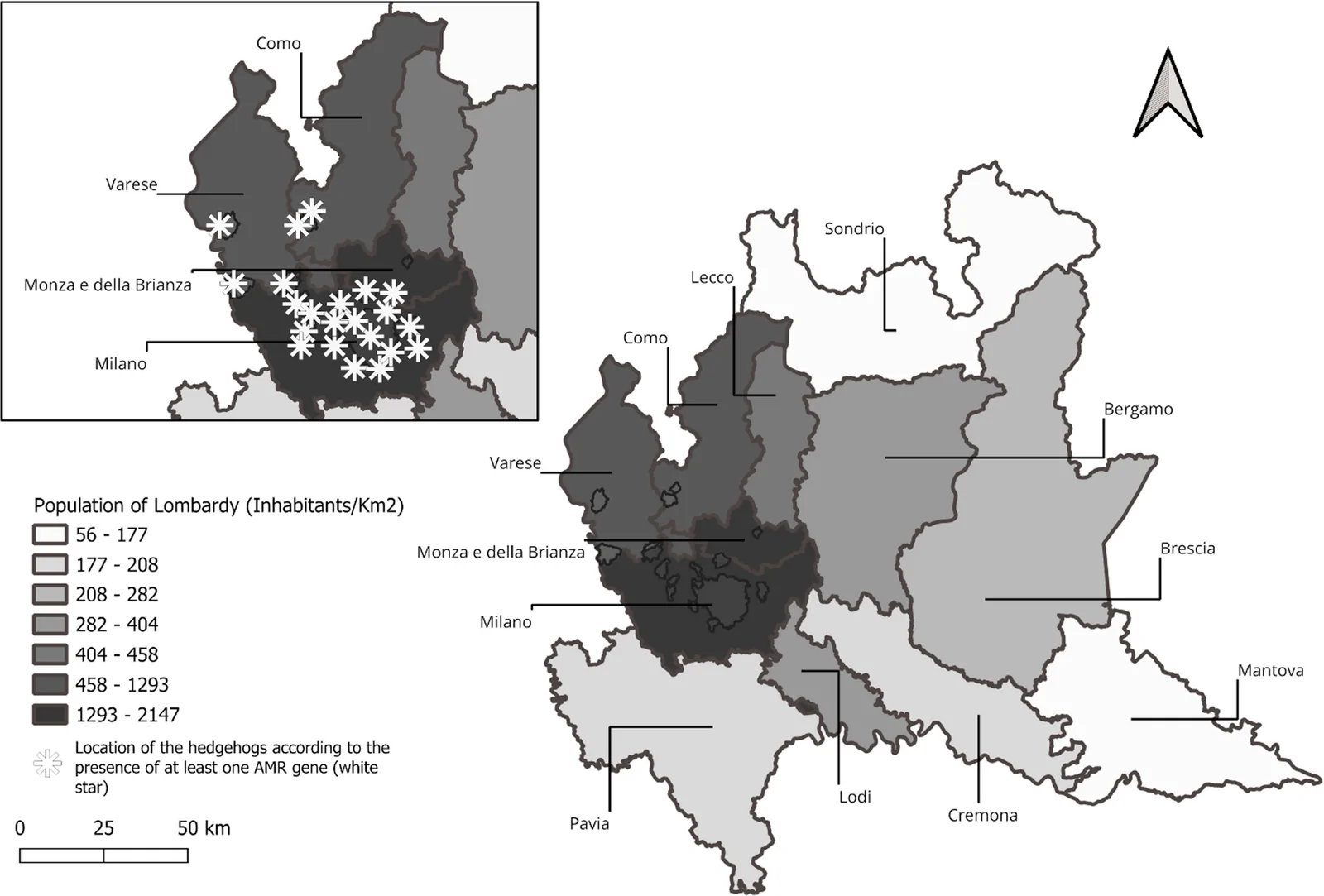
Distribution of the hedgehogs positive for the presence of 3GCR-*E. coli* (carrying at least one AMR gene) in Lombardy region

A total of 49 samples of intestinal content collected from European hedgehogs were tested. These animals originated from the provinces of Milan, Varese, Como, and Monza Brianza, which are some of the most densely populated areas of Northern Italy (Fig. 1).

Overall, 3GCR-*E. coli* was isolated in 25 out of 49 (51%) samples, whereas in the remaining 19 samples (39%) bacterial species not considered in this study, such as *Klebsiella* spp. and non-3GCR-*E. coli*, were isolated. No growth was observed in the remaining 5 samples (10%).

The majority of the 3GCR-*E. coli* isolates exhibited an MDR profile (*n* = 24/25, 96%), according to the adopted definition. Consistent with the 3GCR phenotype, resistance was recorded against β-lactams cefotaxime, cefazolin, and ampicillin in 100% of isolates (*n* = 25/25), whereas 52% (*n* = 13/25) were also resistant to amoxicillin/clavulanic acid. Resistance against fluoroquinolones (enrofloxacin, flumequine) was found in 92% of isolates (*n* = 23/25), and resistance to tetracyclines was found in 64% of isolates (*n* = 16/25). All the isolates were susceptible to colistin and florfenicol (100%, *n* = 25/25), and high sensitivity was recorded for kanamycin (96%, *n* = 24/25). The MIC results are detailed in Supplementary Materials File S1.

**Fig. 2:**
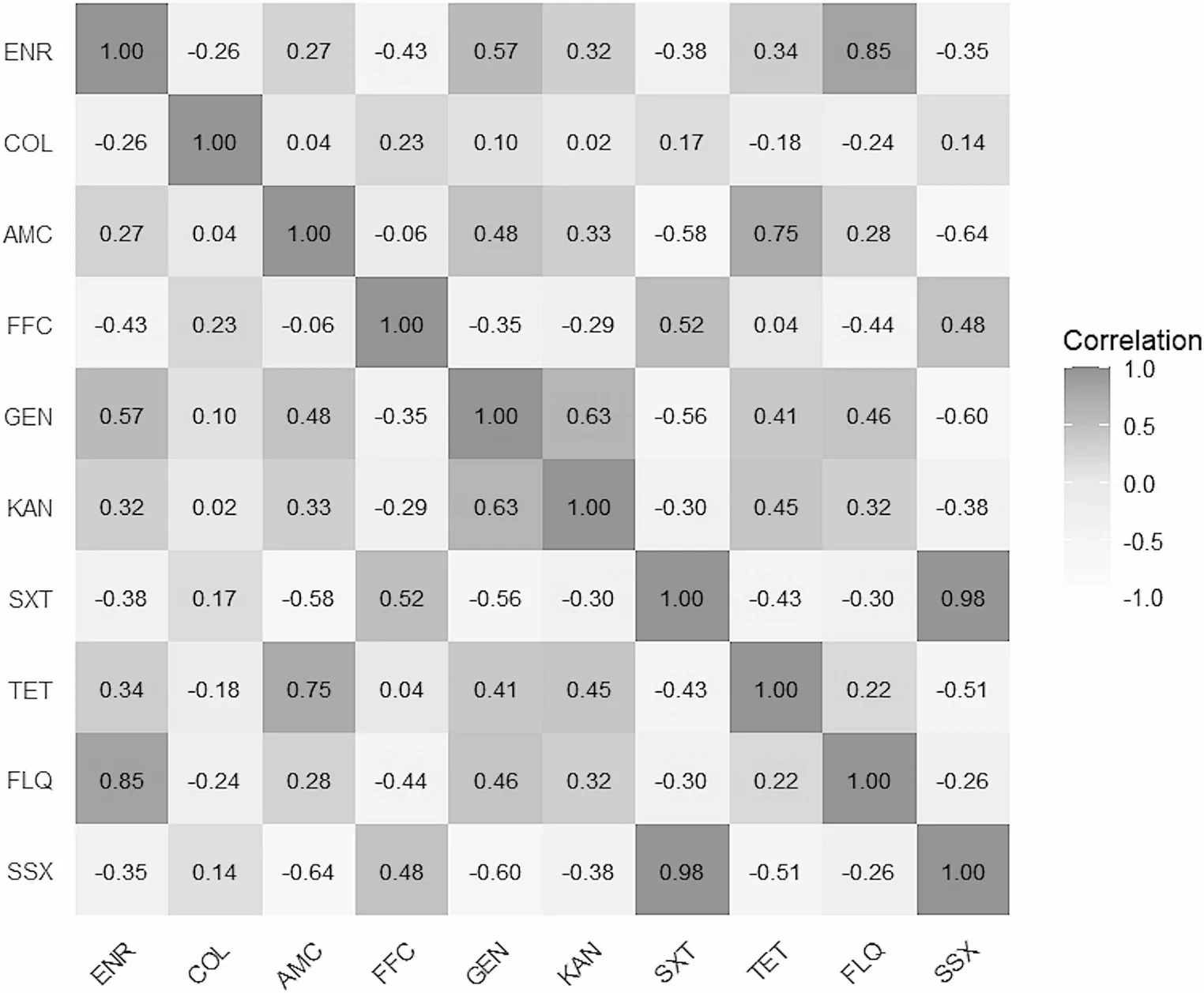
Spearman correlation matrix of raw MIC values (treated as ordinal data) among the antimicrobial molecules tested in 3GCR-*E. coli* isolates. MIC values were analyzed without log2 transformation and without conversion into binary resistance categories. Ampicillin, cefazolin, and cefotaxime were excluded from the analysis because all isolates showed identical MIC values, resulting in no variability. (ENR: enrofloxacin; COL: colistin; AMC: amoxicillin/clavulanic acid; FFC: florfenicol; GEN: gentamicin; KAN: kanamycin; SXT: trimethoprim/sulfamethoxazole; TET: tetracycline; FLQ: flumequine; SSX: sulfisoxazole)

In Table 1 is described the phenotypic and genomic AMR characterization of each isolate.Strong correlations were observed, particularly within antimicrobials belonging to the same class (*p* < 0.01), as for enrofloxacin/flumequine (ρ = 0.85), and trimethoprim-sulfamethoxazole/sulfisoxazole (ρ = 0.98) (Fig. 2).

**Table 1:** Antimicrobial resistance phenotypes and genotypes detected in European hedgehogs (βL-Pen: penicillins (amoxicillin/clavulanic acid, ampicillin); β-Cep: cephalosporins (cefazolin, cefotaxime); FQ: Fluoroquinolones (enrofloxacin, flumequine); AG: aminoglycosides (gentamycin, kanamycin); TET: tetracyclines; SUL: sulphonamides (sulfisoxazole, trimethoprim-sulfamethoxazole)

Samples	AMR phenotyping	β-lactam resistance genes
1	βL-Pen, β-Cep, FQ, TET	TEM
2	βL-Pen, β-Cep, FQ	SHV
3	βL-Pen, β-Cep	CTX-M
4	βL-Pen, β-Cep, FQ, AG, TET	CTX-M
5	βL-Pen, β-Cep, FQ, TET	TEM, SHV
6	βL-Pen, β-Cep, FQ, TET	TEM, SHV
7	βL-Pen, β-Cep, FQ, TET	TEM, SHV
8	βL-Pen, β-Cep, FQ, TET	TEM, SHV
9	βL-Pen, β-Cep, FQ, AG, TET	TEM, CTX-M
10	βL-Pen, β-Cep, FQ, AG, TET	TEM, CTX-M
11	βL-Pen, β-Cep, SUL, TET	None
12	βL-Pen, β-Cep, FQ, SUL	None
13	βL-Pen, β-Cep, FQ, TET	TEM, SHV
14	βL-Pen, β-Cep, FQ, AG	CTX-M
15	βL-Pen, β-Cep, FQ, SUL	CTX-M
16	βL-Pen, β-Cep, FQ, SUL	None
17	βL-Pen, β-Cep, FQ, AG, TET	CTX-M
18	βL-Pen, β-Cep, FQ, SUL	SHV
19	βL-Pen, β-Cep, FQ, TET	TEM
20	βL-Pen, β-Cep, FQ, TET	None
21	βL-Pen, β-Cep, FQ, TET	None
22	βL-Pen, β-Cep, FQ, SUL, TET	TEM
23	βL-Pen, β-Cep, FQ, TET	TEM
24	βL-Pen, β-Cep, FQ, SUL	SHV
25	βL-Pen, β-Cep, FQ, SUL	SHV

**Fig. 3:**
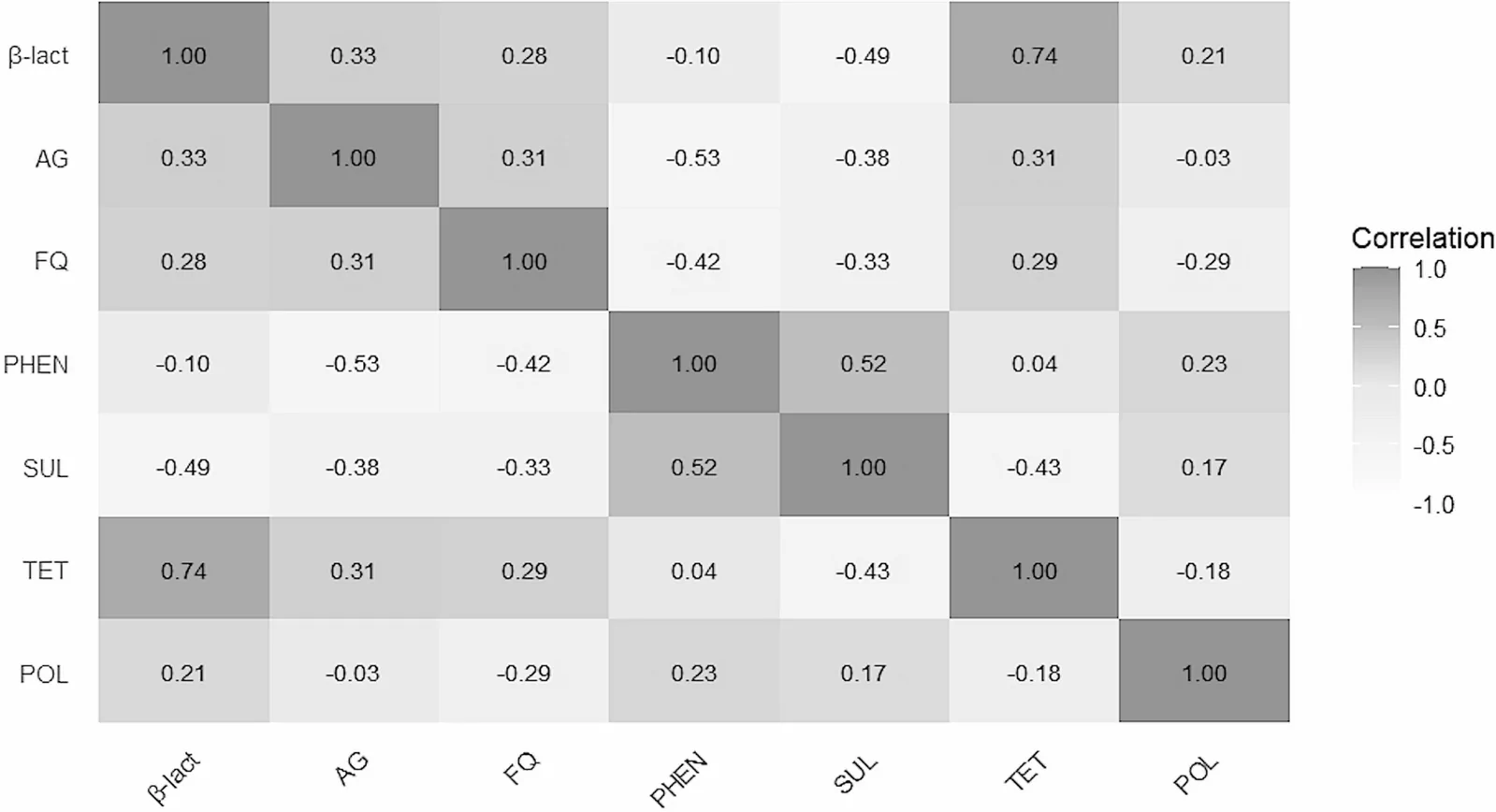
Spearman correlation matrix between antimicrobial classes based on raw MIC data, treated as ordinal values, without log2 transformation and without conversion into binary resistance categories. Antimicrobials showing no MIC variability among isolates were excluded from the analysis. (β-lact: β-lactams; AG: aminoglycosides; FQ: fluoroquinolones; PHEN: phenicols; SUL: sulphonamides; TET: tetracyclines; POL: polymyxins)

At the antimicrobial class level, correlations were observed between β-lactams/tetracyclines (ρ = 0.74) and sulphonamides/phenicols (ρ = 0.52). No correlations were found between β-lactams/sulphonamides (ρ= − 0.49), and between aminoglycosides/phenicols (ρ= − 0.53) (Fig. 3).

Regarding the genetic determinants of resistance, *bla*_TEM_ was the most frequently detected gene (52%, *n* = 13/25), followed by *bla*_SHV_ (36%, *n* = 9/25), and *bla*_CTX−M_ (28%, *n* = 7/25). Seven isolates harbored 2 genes: 5 carried *bla*_TEM_/*bla*_SHV_, and 2 carried *bla*_TEM_/*bla*_CTX−M_ (Table 1). No isolate carried *bla*_CMY−2_. Table 2 reports the number of isolates tested, the frequency distribution of MIC values across the tested dilution range (0.015 – ≥512), and MIC_50_ and MIC_90_ for each molecule.

**Table 2:** Number of isolates tested (#) with broth dilution and distribution of MIC values for each antibiotic. MIC50 and MIC90 are also reported for each molecule. Breakpoints for each antibiotic, indicated by the black bars, mark the concentration thresholds used to categorize isolates as susceptible or resistant. The grey areas indicate the dilutions that were not tested

Antibiotic	#	≤ 0.015	0.03	0.06	0.12	0.25	0.5	1	2	4	8	16	32	64	128	256	≥ 512	MIC50	MIC90
Colistin	25					12	13											0.5	0.5
Amoxicillin/Clavulanic Acid	25								2	6	4		13					32	32
Ampicillin	25												25					32	32
Cefazolin	25										25							8	8
Cefotaxime	25									25								4	4
Gentamicin	25						8	5	7				5					1	32
Kanamycin	25								8	7	8	1	1					4	8
Enrofloxacin	25		1	1			9	8	1			1	4					1	32
Flumequine	25							2		8	10	5						8	16
Florfenicol	25									5	18	2						8	8
Trimethoprim/Sulfamethoxazole	25			17		4						4						0.06	16
Sulfisoxazole	25														17		8	128	512
Tetracycline	25							2	6	1		16						16	16

## Discussion

The results of this study contribute to the growing body of evidence showing that wildlife, including synanthropic species such as the European hedgehog (*Erinaceus europaeus*), can carry ARB and may be useful for environmental AMR surveillance (Carroll et al. 2015; Swift et al. 2019; Skarzynska et al. 2020). In this study, 49 European hedgehogs were sampled, all of which died without receiving any antimicrobial treatment during their stay at the wildlife rescue center. The decision to monitor *E. coli* was based on its ubiquitous nature and its ability to acquire and spread ARGs through both vertical transfer and HGT (Holmes et al. 2016; Von Wintersdorff et al. 2016). We investigated the AMR profile of *E. coli* isolates through a phenotypical (MIC) and genomic approach (ARGs detection) to provide valuable insights into the distribution and spread of AMR in the study area.

Microbiological analysis showed that 51% (*n* = 25/49) of the samples yielded 3GCR-*E. coli* on selective medium. Significant positive correlations were observed between antimicrobials belonging to the same class or sharing related resistance patterns. In particular, strong correlations (*p* < 0.01) were found between the quinolones enrofloxacin and flumequine (Spearman’s rho = 0.85), and between sulfisoxazole and trimethoprim/sulfamethoxazole (Spearman’s rho = 0.98). These conditions are not unexpected and likely reflect shared mechanisms of action and resistance. Therefore, these findings should be interpreted as internal consistency of the dataset rather than evidence of novel biological associations. The 96% (*n* = 24/25) of the 3GCR-*E. coli* isolates exhibited a MDR profile,, with particularly high resistance to β-lactams (100%, *n* = 25/25), especially cefotaxime, cefazolin, and ampicillin, followed by flumequine (quinolones) (92%, *n* = 23/25, and tetracyclines (64%, *n* = 16/25). Resistance to major antibiotic classes such as β-lactams, fluoroquinolones, and tetracyclines is consistent with previous findings in both wildlife and domestic animals, which could potentially reflect selective pressures from environmental contamination. While not directly tested in this study, these patterns are consistent with hypotheses regarding exposure to anthropogenic sources of contamination, particularly in urban and agricultural environments (Vittecoq et al. 2016). The presence of resistance to third generation cephalosporins in wildlife-associated *E. coli* isolates is concerning, given the critical role of cephalosporins in human medicine and their classification by the World Health Organization (WHO) as “Critically Important Antibiotics (CIAs)” in human medicine (World Health Organization 2024). Regarding tetracycline resistance, the observed prevalence (64%, *n* = 16/25) is in line with the findings reported by Garcias and colleagues (2021). Tetracyclines have been widely used in animal husbandry for decades, mainly for growth promotion and disease prevention in livestock. This extensive use of this class, along with their persistence in the environment, facilitated the selection and spread of ARB in agricultural and urban ecosystems (Manyi-Loh et al. 2018). Notably, MIC analysis revealed a moderate correlation between β-lactams and tetracyclines (Spearman coefficient = 0.74), raising the hypothesis of a potential co-selection mechanism. Additional associations included weak correlations between β-lactams and aminoglycosides (0.33), and between sulphonamides and phenicols (0.52), as well as negative correlations between β-lactams and sulphonamides (–0.49), and between aminoglycosides and phenicols (–0.53). These findings suggest that hedgehogs admitted to rescue facilities may provide useful information on environmental AMR patterns, particularly in densely populated or intensively farmed areas. However, given the opportunistic rescue-centre-based sampling design, no inference can be made about the occurrence of these resistance patterns in the wider free-ranging hedgehog population. In the Italian context, AMR *E. coli* has been documented in several wildlife taxa and ecological settings, supporting the relevance of wildlife-based surveillance within a One Health framework. In wild birds admitted to a wildlife rescue centre in Turin, *E. coli* isolates frequently showed MDR (39.6%) and Extended Spectrum β -lactamase (ESBL) production (13.2%), with resistance levels increasing during hospitalization, whereas in hunted wild boar from Tuscany high resistance rates were reported especially to cephalothin, amoxicillin/clavulanic acid, ampicillin, and tetracycline (Bertelloni et al. 2020; Prandi et al. 2023). Likewise, in Maiella National Park, resistant bacteria, including *E. coli*, were recovered from wildlife, livestock, and water, and samples from sympatric wildlife-livestock areas were more likely to contain resistant isolates (Smoglica et al. 2023). More recently, in Northern Italy, Rapi et al. 2025 found that *E. coli* was the most frequently isolated Gram-negative species in wild birds examined at a wildlife rescue centre in Lombardy, with the highest resistance rates observed against piperacillin, ampicillin, and tetracycline, alongside additional resistance to fluoroquinolones, chloramphenicol, sulphonamides, and colistin in a subset of isolates (Rapi et al. 2025). Alongside whit these studies, in the national context evidence specifically focused on AMR *E. coli* isolated from European hedgehogs is still limited but growing. A recent Italian study on hedgehogs admitted to a wildlife rescue centre in Turin showed that animals could already harbor resistant *E. coli* at admission, particularly against cefazolin, ampicillin, and enrofloxacin, with ESBL-producing isolates also detected and increasing during hospitalization (Prandi et al. 2025). In addition, other bacterial targets have been investigated in this host: *Salmonella* spp. were isolated from 30% of hedgehogs sampled in rehabilitation centres across four Italian regions, although most isolates were fully susceptible, while antimicrobial-resistant *Staphylococcus* spp. were also documented in hedgehogs from central Italy, including MDR and XDR profiles (Bertelloni et al. 2025; Barbarulo et al. 2026).

At the genetic level, our findings are only partially consistent with previous reports, suggesting differences in the distribution of AMR determinants across wildlife species and study settings. Torres et al. (2022) found *bla*_TEM_ to be the most prevalent ESBL-coding gene in wild ungulates, with 85% of isolates harboring this determinant, while *bla*_CTX−M_ was detected at a much lower rate (8.5%). In contrast, Garcias et al. (2021) reported a higher prevalence of *bla*_CTX−M_ in wild hedgehogs, highlighting potential differences in selective pressures across urban-adapted and rural wildlife populations. Moreover, Darwich and colleagues (2019) reported *bla*_CTX−M_ as the most frequent β-lactamase resistance gene in wildlife-associated *E. coli*, whereas they did not detect *bla*_TEM_. In our study, PCR screening identified β-lactamase-related targets in a proportion of isolates, with *bla*_TEM_ being the most frequently detected gene family (52%, *n* = 13/25), followed by *bla*_SHV_ (36%, *n* = 9/25), and *bla*_CTX−M_ (28%, *n* = 7/25). Furthermore, 7 isolates carried 2 β-lactamase-related targets, including 5 with *bla*_TEM_/*bla*_SHV_, and 2 with *bla*_TEM_/*bla*_CTX−M_. The discrepancies in the distribution of genetic determinants suggest that environmental exposure, host species differences, and regional antibiotic usage may significantly influence the frequency and dissemination of ARGs. However, these results should be interpreted with caution, since the molecular analysis was designed as a targeted screening approach rather than a variant-level characterization, and positive amplicons were not sequenced. Therefore, *bla*_TEM_ and *bla*_SHV_ were identified only at the gene-family level, and it was not possible to distinguish ESBL from non-ESBL variants within these families. In light of this limitation, the observed distribution of genetic determinants should be considered indicative of the circulation of β-lactamase-associated resistance targets rather than definitive evidence of specific ESBL variants. Further sequencing-based characterization would be useful to better define the molecular resistance profile of these isolates. 

The detection of AMR bacteria in the hedgehogs examined in this study may have ecological and epidemiological relevance, particularly because this species frequently occurs in urban and peri-urban environments and can come into contact with anthropogenic waste, sewage-contaminated water, and domestic animals (Garcias et al. 2021). Furthermore, the observation that resistant isolates showed significant correlations within antimicrobial classes (e.g. quinolones/fluoroquinolones, sulphonamides) suggests strong selective pressure, likely due to environmental contamination (Vittecoq et al. 2016). Although our study did not include direct environmental or human sampling, these correlations suggest a possible strong selective pressure. Such patterns might be of particular concern in human-impacted environments, where hedgehogs could potentially be exposed to ARB from multiple environmental source. Previous studies have shown that urban wildlife often harbors ARB closely related to clinical isolates, suggesting a bidirectional exchange between humans and wildlife (Darwich et al. 2019). Some of the antimicrobials tested are classified by the World Health Organization (WHO) (World Health Organization 2024) as CIAs and “Highest priority critically important antimicrobials” (HPCIA) for human medicine, while according to the European Medicines Agency several fall within Category B (“Restrict”) or A (“Avoid”) for veterinary use (EMA 2025). Although based on different criteria, both classification systems highlight their relevance for public health. However, resistance to these molecules has also been reported in *E. coli* isolates from livestock and wildlife, including birds and ungulates (deer, roe deer, and wild boar) (Wasyl et al. 2018; Torres et al. 2020; Rapi et al. 2025).

The present findings support inclusion of wildlife in AMR surveillance frameworks. In particular, European hedgehogs admitted to wildlife rescue facilities may provide useful, although non-representative, information on the occurrence of AMR *E. coli* in human-impacted environments. In this study, 3GCR-*E. coli* were frequently detected and the majority of them showed MDR phenotypes. While these findings support the hypothesis that hedgehogs may serve as sentinels for environmental AMR, we emphasize that without direct comparative data from external AMR sources, the exact drivers and pathways of this resistance remain to be elucidated. Also, because the animals examined were obtained through an opportunistic rescue-centre-based sampling design, and not through a field-based survey, the results cannot be generalized to the wider free-ranging hedgehog population in Lombardy. Nevertheless, our data support a conservative interpretation of the European hedgehog as a potential sentinel species for environmental AMR, and the geographical focus of the present study fills a significant gap in the existing literature, as most previous studies on AMR in wildlife have been conducted in other European regions, leaving a paucity of data from Northern Italy.

It is however important to acknowledge some limitations of this study. First, the lack of concurrent sampling from the surrounding environment, livestock, or human populations prevents a definitive identification of the sources of the ARGs detected. Second, without comparative genomic analysis or whole-genome sequencing (WGS), the hypothesized bidirectional exchange between humans and wildlife remains speculative, albeit supported by the ecological context of the sampling area. Furthermore, only one *E. coli* isolate per positive sample was selected for antimicrobial susceptibility testing and molecular analysis. While this approach is commonly adopted for standardization purposes, it may underestimate the within-host diversity and fail to capture the coexistence of multiple strains with different antimicrobial resistance profiles within the same individual. Future studies should consider the analysis of multiple colonies per sample and the application of high-resolution approaches, such as WGS, to better characterize intra-host diversity and resistance dynamics between wildlife-derived *E. coli* isolates and those found in human or livestock-associated reservoirs (Torres et al. 2020). In addition, future research should focus on elucidating the mechanisms underlying AMR acquisition in hedgehogs and other urban wildlife species, while prioritizing field-based and systematically designed surveys in order to assess the occurrence of AMR in free-ranging hedgehog populations. Longitudinal studies investigating the temporal trends of AMR in wildlife populations, combined with metagenomic analysis of environmental samples, could provide valuable insights into the sources and drivers of resistance gene spread. Although data on geographical origin, cause of admission, and time spent in care were collected, the limited sample size and heterogeneity of these variables did not allow for a meaningful stratified analysis. Future studies with larger and more balanced datasets should investigate these factors to better understand potential epidemiological drivers of AMR in hedgehogs. Additionally, animals admitted to wildlife rescue centers may not fully represent truly free-ranging populations, which should be considered when interpreting the results.

## Supplementary Information

Below is the link to the electronic supplementary material.

Supplementary Material 1

## Data Availability

All data generated or analysed during this study are included in this published article and its supplementary information files.
