# Supplementary material for: Occurrence of third-generation cephalosporin-resistant Escherichia coli in European hedgehogs (Erinaceus europaeus) from a wildlife rescue centre in Lombardy, Northern Italy
Source: Vet Res Commun. 2026 Jun 20;50(5):410. doi: 10.1007/s11259-026-11356-4 (PMC13283182; doi:10.1007/s11259-026-11356-4)
Supplement: Supplementary file 1 — Supplementary Material 1 [file 11259_2026_11356_MOESM1_ESM.docx]

Supplementary File S1. MIC values expressed in μg/mL for the ESBL-producing *E. coli*. (R: resistant; S: susceptible).

| Isolate | Colistin | | Amoxicillin/  Clavulanic Acid | | Ampicillin | | Cefazolin | | Cefotaxime | | Gentamicin | | Kanamycin | | Enrofloxacin | | Flumequine | | Florfenicol | | Trimethoprim/  Sulfamethoxazole | | Sulfisoxazole | | Tetracycline | |
| --- | --- | --- | --- | --- | --- | --- | --- | --- | --- | --- | --- | --- | --- | --- | --- | --- | --- | --- | --- | --- | --- | --- | --- | --- | --- | --- |
| 1 | 0.25 | S | 32 | R | 32 | R | 8 | R | 4 | R | 1 | S | 2 | S | 1 | R | 8 | R | 8 | S | 0.06 | S | ≤128 | S | 16 | R |
| 2 | 0.25 | S | 4 | S | 32 | R | 8 | R | 4 | R | 0.5 | S | 4 | S | 0.5 | R | 4 | R | 4 | S | 0.06 | S | ≤128 | S | 1 | S |
| 3 | 0.5 | S | 8 | S | 32 | R | 8 | R | 4 | R | 2 | S | 4 | S | 0.06 | S | 1 | S | 8 | S | 0.06 | S | ≤128 | S | 2 | S |
| 4 | 0.25 | S | 4 | S | 32 | R | 8 | R | 4 | R | 32 | R | 32 | R | 32 | R | 16 | R | 8 | S | 0.06 | S | ≤128 | S | 16 | R |
| 5 | 0.25 | S | 32 | R | 32 | R | 8 | R | 4 | R | 2 | S | 2 | S | 1 | R | 8 | R | 8 | S | 0.06 | S | ≤128 | S | 16 | R |
| 6 | 0.25 | S | 32 | R | 32 | R | 8 | R | 4 | R | 1 | S | 2 | S | 1 | R | 8 | R | 8 | S | 0.06 | S | ≤128 | S | 16 | R |
| 7 | 0.25 | S | 32 | R | 32 | R | 8 | R | 4 | R | 2 | S | 8 | S | 1 | R | 8 | R | 8 | S | 0.06 | S | ≤128 | S | 16 | R |
| 8 | 0.25 | S | 32 | R | 32 | R | 8 | R | 4 | R | 0.5 | S | 4 | S | 1 | R | 8 | R | 8 | S | 0.06 | S | ≤128 | S | 16 | R |
| 9 | 0.25 | S | 32 | R | 32 | R | 8 | R | 4 | R | 32 | R | 8 | S | 32 | R | 16 | R | 4 | S | 0.06 | S | ≤128 | S | 16 | R |
| 10 | 0.25 | S | 32 | R | 32 | R | 8 | R | 4 | R | 32 | R | 16 | S | 32 | R | 16 | R | 4 | S | 0.06 | S | ≤128 | S | 16 | R |
| 11 | 0.25 | S | 4 | S | 32 | R | 8 | R | 4 | R | 0.5 | S | 8 | S | 0.03 | S | 1 | S | 8 | S | 16 | R | >512 | R | 16 | R |
| 12 | 0.25 | S | 2 | S | 32 | R | 8 | R | 4 | R | 0.5 | S | 2 | S | 1 | R | 8 | R | 8 | S | 0.25 | S | >512 | R | 2 | S |
| 13 | 0.5 | S | 8 | S | 32 | R | 8 | R | 4 | R | 2 | S | 4 | S | 2 | R | 4 | R | 8 | S | 0.06 | S | ≤128 | S | 16 | R |
| 14 | 0.5 | S | 8 | S | 32 | R | 8 | R | 4 | R | 32 | R | 8 | S | 16 | R | 16 | R | 4 | S | 0.06 | S | ≤128 | S | 2 | S |
| 15 | 0.5 | S | 4 | S | 32 | R | 8 | R | 4 | R | 0.5 | S | 2 | S | 0.5 | R | 4 | R | 8 | S | 16 | R | >512 | R | 4 | S |
| 16 | 0.25 | S | 2 | S | 32 | R | 8 | R | 4 | R | 0.5 | S | 2 | S | 0.5 | R | 4 | R | 8 | S | 0.25 | S | >512 | R | 2 | S |
| 17 | 0.5 | S | 32 | R | 32 | R | 8 | R | 4 | R | 32 | R | 8 | S | 32 | R | 16 | R | 4 | S | 0.06 | S | ≤128 | S | 16 | R |
| 18 | 0.5 | S | 4 | S | 32 | R | 8 | R | 4 | R | 0.5 | S | 2 | S | 1 | R | 8 | R | 8 | S | 0.25 | S | >512 | R | 2 | S |
| 19 | 0.5 | S | 32 | R | 32 | R | 8 | R | 4 | R | 1 | S | 8 | S | 0.5 | R | 8 | R | 8 | S | 0.06 | S | ≤128 | S | 16 | R |
| 20 | 0.5 | S | 32 | R | 32 | R | 8 | R | 4 | R | 1 | S | 4 | S | 0.5 | R | 4 | R | 8 | S | 0.06 | S | ≤128 | S | 16 | R |
| 21 | 0.5 | S | 32 | R | 32 | R | 8 | R | 4 | R | 2 | S | 8 | S | 0.5 | R | 4 | R | 8 | S | 0.06 | S | ≤128 | S | 16 | R |
| 22 | 0.5 | S | 32 | R | 32 | R | 8 | R | 4 | R | 1 | S | 8 | S | 1 | R | 8 | R | 16 | S | 16 | R | >512 | R | 16 | R |
| 23 | 0.5 | S | 32 | R | 32 | R | 8 | R | 4 | R | 2 | S | 4 | S | 0.5 | R | 4 | R | 8 | S | 0.06 | S | ≤128 | S | 16 | R |
| 24 | 0.5 | S | 4 | S | 32 | R | 8 | R | 4 | R | 0.5 | S | 2 | S | 0.5 | R | 8 | R | 8 | S | 0.25 | S | >512 | R | 1 | S |
| 25 | 0.5 | S | 8 | S | 32 | R | 8 | R | 4 | R | 2 | S | 4 | S | 0.5 | R | 4 | R | 16 | S | 16 | R | >512 | R | 2 | S |
